# Weekday and outcomes of elective cardiac surgery in the UK: a large retrospective database analysis

**DOI:** 10.1093/ejcts/ezac038

**Published:** 2022-01-29

**Authors:** Daniel Paul Fudulu, Arnaldo Dimagli, Shubhra Sinha, Pradeep Narayan, Jeremy Chan, Tim Dong, Umberto Benedetto, Gianni Davide Angelini

**Affiliations:** 1 Department of Cardiac Surgery, Bristol Heart Institute, University of Bristol, Bristol, UK; 2 Department of Cardiac Surgery, Rabindranath Tagore International Institute of Cardiac Sciences, Kolkata, India

**Keywords:** Outcomes, Seasonality, Day of the week, Cardiac surgery, The weekend effect

## Abstract

**OBJECTIVES:**

Several studies have shown worse outcomes in patients operated on later in the week. We tested this hypothesis in a large UK national audit database in elective patients undergoing adult cardiac surgery.

**METHODS:**

We used a generalized additive model to evaluate the effect of the day of the week on the following postoperative outcomes: 30-day mortality, stroke, need for dialysis and return to theatre for bleeding. We have adjusted for the relevant European System for Cardiac Operative Risk Evaluation (EuroSCORE) II covariates, plus responsible consultant, hospital and year of operation and performed subgroup analysis for isolated coronary artery bypass grafting (CABG) procedures.

**RESULTS:**

Out of 371 500 patients, 60 555 (16.3%) underwent AVR, 36 553 (9.8%) AVR plus CABG, 238 812 (64.3%) isolated CABG, 26 517 (7.1%) isolated mitral valve repair or replacement and 9063 (2.4%) mitral valve plus CABG. A total of 13 997 (3%) had surgery over the weekend. After covariate adjustment, we found no effect of day of surgery on mortality (*P* = 0.081), stroke (*P* = 0.137) and need for postop dialysis (*P* = 0.732). However, across all operations, there was evidence of a lower rate of return to theatre for bleeding/tamponade at the weekend (*P* = 0.039). In subgroup analysis of isolated CABG, the day of the week did not affect any outcomes.

**CONCLUSIONS:**

We found no effect of the day of the week on risk-adjusted short-term mortality, stroke, and the requirement for postoperative dialysis after elective cardiac surgery. Overall, the patients operated on during the weekdays were less likely to return to theatre for bleeding. In isolated CABG, the day of the week did not affect any outcomes.

**Figure:**
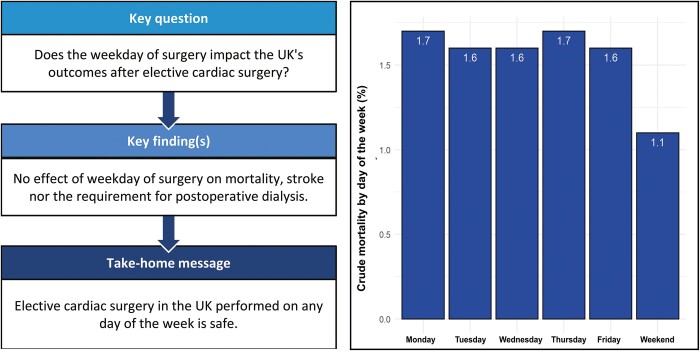

## INTRODUCTION

More than a decade ago, several studies argued for a higher mortality rate for weekend admissions [1–4]. This potential weekday variation on outcomes has also been investigated in patients undergoing surgery. This body of evidence from different countries shows conflicting results. One very large UK study of hospital episode statistics on over 4 million patients admitted for elective surgery showed a higher risk of death in patients who were operated on later in the week [5]. Other studies have shown conflicting results in patients undergoing surgery in general [2, 6, 7]. Several studies have investigated the weekday seasonality of outcomes in cardiac surgery [8–10] and found no effect of the day of surgery on outcomes. It is hypothesized that a potential weekday variation in cardiac surgery outcomes could be related either to more inferior quality of care in certain days of the week due to a shortage of resources or due to surgeon fatigue or experience that could influence performance later in the week [10]. We performed a very large UK adult cardiac surgery database retrospective analysis in elective patients undergoing cardiac surgery to test if the day of the week influences outcomes after elective.

## METHODS

### Ethics statement

The study was approved by the Health Research Authority and Health and Care Research Wales, and a waiver for patients' consent was obtained (IRAS ID: 278171).

### Patients

**Figure 1:**
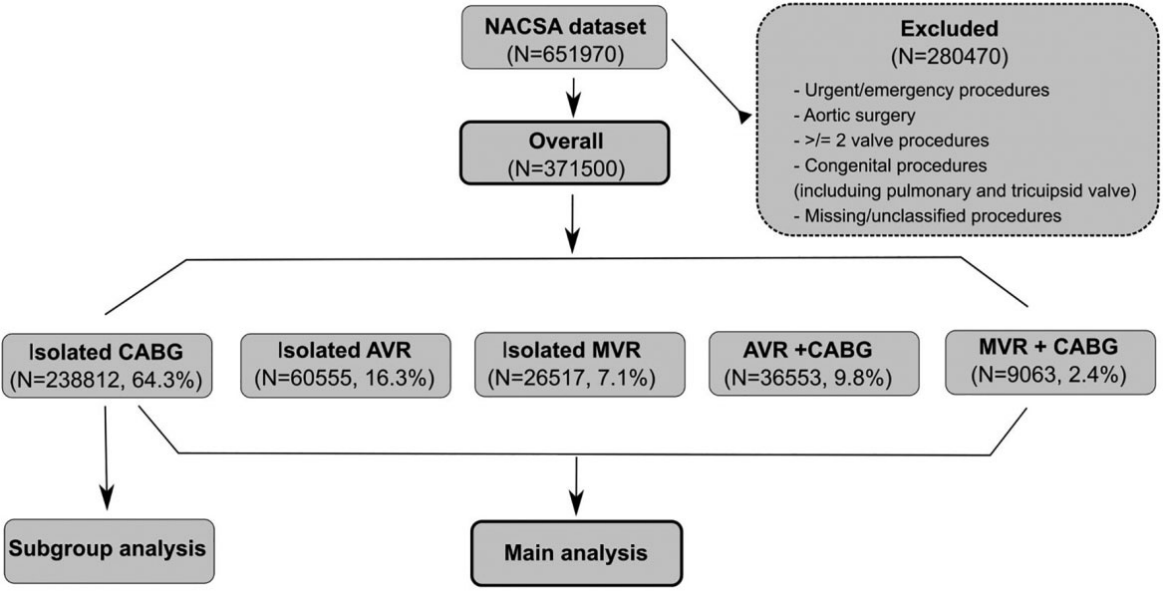
Flow diagram of the procedure groups selected for the analysis.

The National Adult Cardiac Surgery Audit registry prospectively collects demographic and pre-, peri- and postoperative clinical information, including mortality, for all significant adult cardiac surgery procedures performed in the UK. The main role of this registry is to benchmark surgical practice. Operations were performed between February 1996 and March 2019. To focus on a homogeneous population from a procedural risk point of view, we looked at isolated aortic valve replacement, isolated coronary artery bypass grafting (CABG), isolated mitral valve repair/replacement (MVR) and combined procedures (aortic valve replacement plus CABG or MVR plus CABG). We excluded: emergency or urgent operation, redo operations, aortic surgery, congenital procedures, tricuspid valve surgery, pulmonary valve surgery and included single valve procedures only (aortic and mitral with or without CABG) (Fig. 1).

It has been shown that urgent admissions are, on average, fewer at the weekend [11]. One possible reason is that urgent patients who would have presented over the weekend are admitted later in the week (Monday/Tuesday). Assuming that urgent patients who might otherwise be admitted on the weekend present later in the this will affect the weekday mortality rather than the weekend outcomes. Furthermore, we could assume that these urgent patients might not receive timely treatment, resulting in higher mortality. This could influence potential differences between weekday and weekend mortality. To eliminate this potential source of bias in the analysis of seasonality of outcomes, we decided to focus on elective cases only similar to other groups [5].

Moreover, we have performed a subgroup analysis on the most commonly performed procedure-isolated CABG procedures (Table 4 and Fig. 4). We decided to perform a subanalysis of the CABG subgroup because this is also one of the most commonly performed procedures in adult cardiac surgery.

### Statistical analysis

Categorical variables were summarized as counts and percentages. Continuous variables were summarized as median and interquartile range. A Shapiro–Wilks test was used to assess the normality of the distribution of continuous data. Data of normal distribution were averaged as a mean with standard deviation and analysed using a Student's *t*-test. Non-normally distributed data were averaged as a median with interquartile range (IQR) and analysed using a rank sum test. Categorical data are presented as frequencies and compared using a Chi-squared test. Confounders considered included: age, gender, neurological dysfunction, renal dysfunction, recent myocardial infarction (MI), pulmonary disease, unstable angina Canadian Cardiovascular Society (CCS) grading scale 4, New York Heart Association Functional Classification class, pulmonary hypertension (HTN), diabetes on insulin, peripheral vascular disease, procedure type (non-CABG), consultant code and hospital centre. Because the study spans over a long period whereby both procedures and service provision policies could have influenced day of the week outcomes over time, we also have adjusted for the year of the operation.

Due to potential seasonal variation of outcomes and the resulting complex, non-linear interactions, we decided to use a generalized additive model (GAM). These types of models are established in the analysis of seasonality and time series. In contrast to linear models, the dependent variable can be modelled by independent variables in the form of smooth functions (in our case, day of the week). We have used a GAM to evaluate the effect of day of the week on the following outcome variables: 30-day mortality, stroke, need for dialysis and return to theatre for bleeding/tamponade. To investigate weekly seasonality in outcomes, we treated the day of the week as an independent variable modelled by a smoothing function (s), and we used P-splines. We have assessed the significance of the smooth function (*P* < 0.05) applied to the day of the week. Analysis was conducted in R Version 1.4.1106, packages: gtsummary, ggplot2, mgcv, mgcVIZ and sjPlot. Analyses were exploratory in nature, and there was no prespecified plan to adjust for multiple comparisons. The 95% confidence intervals were not adjusted for multiple comparisons, and the inferences drawn from them may not be reproducible. We treated patients operated Saturday and Sunday as 1 group (‘weekend’ group) due to the low number of cases on Saturday and Sunday and because we were interested in assessing a potential overall weekend effect. We have used the mgcv package to run diagnostics for the fitted GAM. A summary of the mgcv model checks is found in Supplementary Material, Table S1.

There was no patient and public involvement in the design of this research or retrospective database analysis.

### Data availability statement

Requests for data should be directed to the lead author (daniel.fudulu@bristol.ac.uk). Requests will be assessed for scientific rigour before being granted. Data will be anonymized and securely transferred. A data-sharing agreement will be required.

## RESULTS

**Figure 2:**
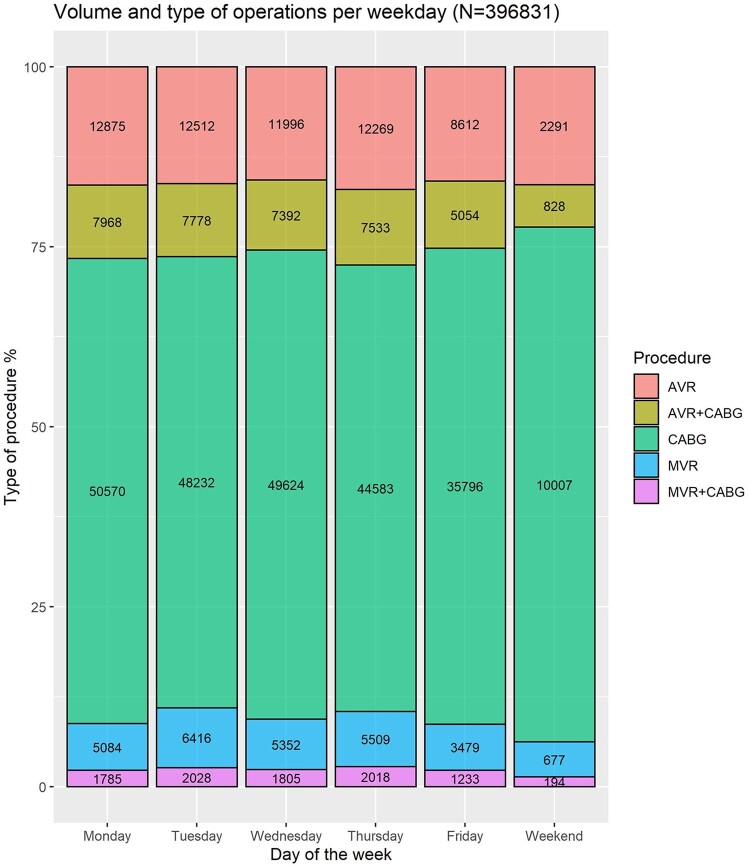
Volume of operations by type of procedures operated on the various days of the week. The *Y*-axis depicts the percentage of procedures. On the bar plots, the absolute number of procedures is depicted.

The volume of all procedures was lower on at the weekend compared to the rest of the week (13.997 cases vs 357.503 cases) (Table 1). The proportions of different types of procedures were relatively similar during the weekday. However, less combined cases (valve and CABG—5.9% vs 10% and MVR ± CABG 1.4% vs 2.5%), less isolated MVRs (4.8% and 7.2%) and more isolated CABG operations (71% vs 64%) were performed at the weekend (Fig. 2). Patients who were operated on over the weekend were younger [median age of 67 (IQR 60, 73) vs median age of 68 (IQR 6, 74), *P* < 0.001] and tended to have fewer comorbidities compared to weekday patients: preop neurological dysfunction (1.5% vs 2.0%, *P* < 0.001), renal dysfunction (0.7% vs 1.3%, *P* < 0.01), previous MI (5.4% vs 6.9%, *P* < 0.01), chronic lung disease (11% vs 12%, *P* = 0.07), unstable angina (4% vs 4.4%, *P* < 0.002), congestive heart failure (1.7% vs 2.5%, *P* < 0.001), diabetes on insulin (4.6% vs 5.5%, *P* < 0.001), peripheral vascular disease (8.2% vs 11%, *P* < 0.001) and left ventricular (LV) dysfunction of any type (*P* < 0.001) (Table 1). This pattern was also consistent for the isolated CABG subgroup (Table 4 and Supplementary Material, Table S3). 

**Table 1:** Baseline characteristics of patients operated by day of the week

Characteristic	Monday, *N* = 78 282^a^	Tuesday, *N* = 76 966^a^	Wednesday, *N* = 76 169^a^	Thursday, *N* = 71 912^a^	Friday, *N* = 54 174^a^	Weekday overall, *N* = 357 503^a^	Weekend, *N* = 13 9971^a^	*P*-Value^b^
Age, median (IQR)	68 (61, 74)	68 (61, 75)	68 (60, 74)	68 (61, 75)	68 (61, 74)	68 (61, 74)	67 (60, 73)	<0.001
Female	19 849 (25)	19 728 (26)	19 220 (25)	18 669 (26)	13 809 (26)	91 275 (26)	3122 (22)	<0.001
Neurological dysfunction	1547 (2.0)	1580 (2.1)	1406 (1.8)	1488 (2.1)	1053 (1.9)	7074 (2.0)	212 (1.5)	<0.001
Creatinine >200 µmol/l	980 (1.3)	1007 (1.3)	1062 (1.4)	1056 (1.5)	719 (1.3)	4824 (1.3)	102 (0.7)	<0.001
Recent MI	5441 (7.0)	5146 (6.7)	5486 (7.2)	4536 (6.3)	4039 (7.5)	24 648 (6.9)	758 (5.4)	<0.001
Pulmonary disease	9131 (12)	8991 (12)	8916 (12)	8579 (12)	6423 (12)	42 040 (12)	1524 (11)	0.017
CCS IV	3368 (4.3)	3357 (4.4)	3416 (4.5)	3083 (4.3)	2576 (4.8)	15 800 (4.4)	560 (4.0)	<0.001
NYHA IV	1833 (2.3)	1885 (2.4)	1871 (2.5)	1844 (2.6)	1349 (2.5)	8782 (2.5)	236 (1.7)	<0.001
Pulmonary HTN	1226 (1.6)	1153 (1.5)	1136 (1.5)	1053 (1.5)	812 (1.5)	5380 (1.5)	265 (1.9)	0.006
Diabetes on insulin	4267 (5.5)	4249 (5.5)	4147 (5.4)	3911 (5.4)	3015 (5.6)	19 589 (5.5)	639 (4.6)	<0.001
LV function								<0.001
Very poor (EF < 20%)	215 (0.3)	201 (0.3)	227 (0.3)	216 (0.3)	145 (0.3)	1004 (0.3)	19 (0.1)	
Poor (EF 21–30%)	838 (1.1)	837 (1.1)	825 (1.1)	800 (1.1)	570 (1.1)	3870 (1.1)	102 (0.7)	
Moderate (EF 31–50%)	7752 (9.9)	7228 (9.4)	7706 (10)	6832 (9.5)	5573 (10)	35 091 (9.8)	1124 (8.0)	
Good (EF > 50%)	69 477 (89)	68 700 (89)	67 411 (89)	64 064 (89)	47 886 (88)	317 538 (89)	12 752 (91)	
Peripheral vascular disease	8223 (11)	8145 (11)	8116 (11)	7568 (11)	5865 (11)	37 917 (11)	1146 (8.2)	<0.001
Consultant first operator	56 118 (79)	55 144 (78)	54 575 (79)	50 926 (78)	38 876 (79)	255 639 (79)	12 023 (94)	<0.001

a *n* (%), median (IQR).

b Pearson's Chi-squared test; Kruskal–Wallis rank sum test (weekday versus weekend).

CABG: coronary artery bypass grafting; EF: ejection fraction; IQR: interquartile range; NYHA: New York Heart Association.

**Figure 3:**
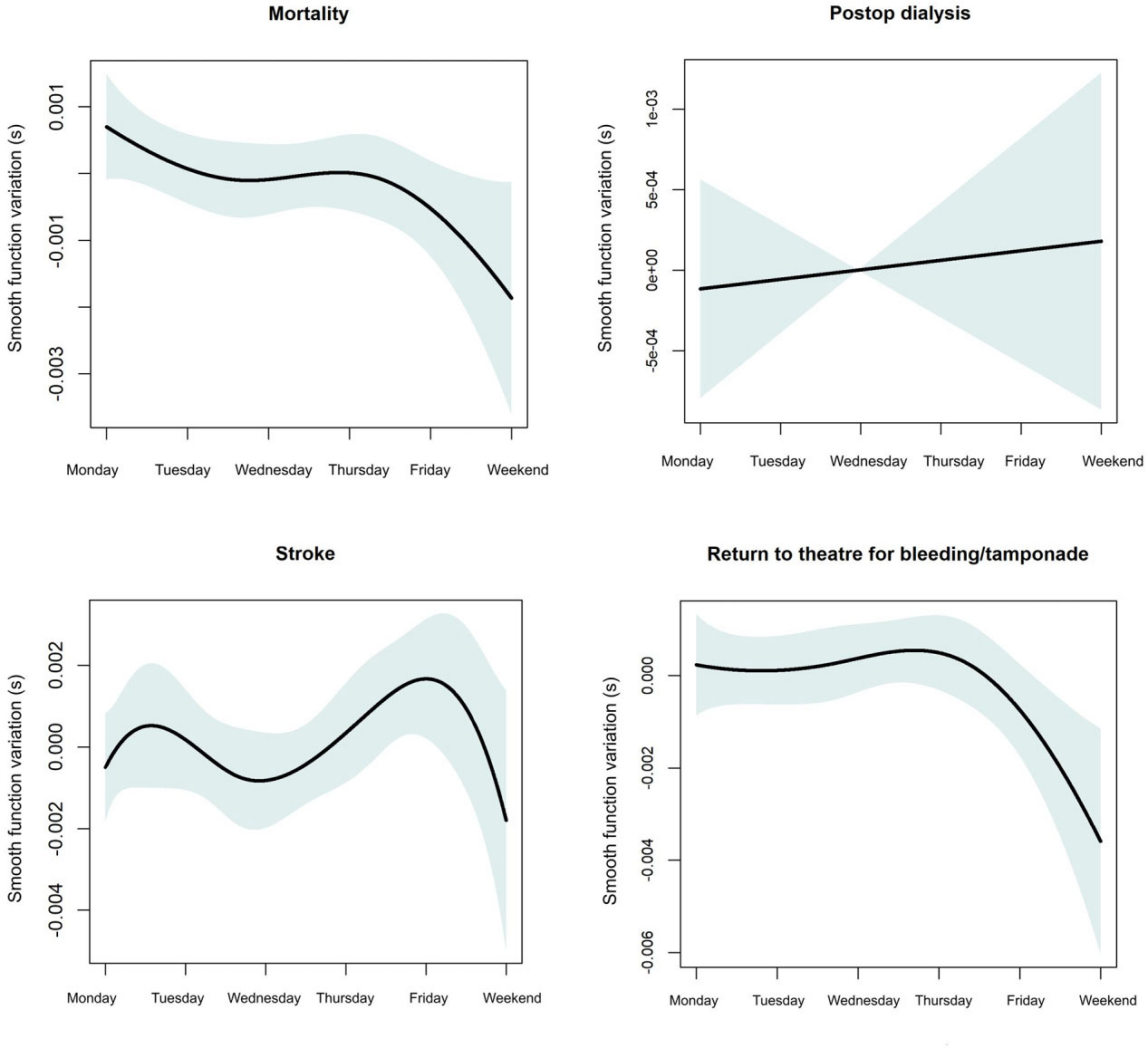
Generalized additive model plots to show the association between the morality, stroke need for pos-top dialysis and return to theatre for bleeding tamponade.

The missing data for mortality were 0.5%, for stroke 12.9%, for the need for postop dialysis 13.7% and for return to theatre for bleeding 3.1%. There were less unadjusted mortality (1.1% vs 1.7%, *P* < 0.001), postop CVA (cerebrovascular accident) without neurological deficit (0.6% vs 0.7%) and with neurological deficit (1.6% vs 2.0%, *P* < 0.002), need for dialysis (1.6% vs 2.0%, *P* < 0.002) and return to the theatre for bleeding (2.6% vs 3.1%, *P* < 0.001) over the weekend compared to the rest of the day of the week (Table 2). After fitting a GAM to the outcomes of interest and correcting for the aforementioned variables, we found no significant effect of the day of the week modelled as the significance of the smooth function on mortality (*P* = 0.081), postoperative CVA (*P* = 0.137) or need for postoperative dialysis (*P* = 0.732). However, there was still significant effect of day of the week on return to theatre for bleeding or tamponade (*P* = 0.039) (Table 3). The smooth plots in Fig. 3 summarize the association between the day of the week and outcomes of interest. While we found no sign of the day of the week on mortality and stroke, it is worth commenting on the patterns in the smooth curves for these outcomes. For mortality, there is a trend towards reduced mortality over the weekend. For stroke, there is a double-hump pattern with peaks in stroke probability early in the week (Monday/Tuesday) and towards the end of the week (e.g. Friday) and dips on Wednesday and the weekend.

**Table 2:** Crude outcomes of patients operated by day of the week

Characteristic	Monday, *N* = 78 282^a^	Tuesday, *N* = 76 966^a^	Wednesday, *N* = 76 169^a^	Thursday, *N* = 71 912^a^	Friday, *N* = 54 174^a^	Weekday overall, *N* = 357 503^a^	Weekend, *N* = 13 997^a^	*P*-Value^b^
Mortality (30 days)	1330 (1.7)	1235 (1.6)	1225 (1.6)	1215 (1.7)	868 (1.6)	5873 (1.7)	148 (1.1)	<0.001
Postop CVA								0.019
CVA without neurological recovery	459 (0.7)	463 (0.7)	434 (0.7)	399 (0.6)	339 (0.7)	2094 (0.7)	71 (0.6)	
CVA with neurological deficit	475 (0.7)	470 (0.7)	454 (0.7)	458 (0.7)	358 (0.8)	2215 (0.7)	69 (0.5)	
Postoperative dialysis	1324 (2.0)	1386 (2.1)	1350 (2.0)	1292 (2.1)	928 (2.0)	6280 (2.0)	204 (1.6)	0.002
Return to theatre for bleeding/tamponade	2417 (3.1)	2451 (3.2)	2391 (3.1)	2267 (3.2)	1659 (3.1)	11 185 (3.1)	363 (2.6)	<0.001

a *n* (%), median (IQR).

b Pearson's Chi-squared test; Kruskal–Wallis rank sum test (weekday versus weekend).

IQR: interquartile range.

**Table 3:** Effect estimates for the EuroSCORE linear predictor and the smooth function (day of the week)

Predictors	Mortality			Postoperative CVA			Postoperative dialysis			Return to theatre for bleeding/tamponade		
	Estimates	CI	*P*-Value	Estimates	CI	*P*-Value	Estimates	CI	*P*-Value	Estimates	CI	*P*-Value
(Intercept)	‒0.03	‒0.08 to 0.02	0.250	‒0.07	‒0.15 to 0.01	0.093	‒0.02	‒0.08 to 0.04	0.585	‒0.04	‒0.11 to 0.03	0.246
Age	0.00	0.00 to 0.00	**<0.001**	0.00	0.00 to 0.00	**<0.001**	0.00	0.00 to 0.00	**<0.001**	0.00	0.00 to 0.00	**<0.001**
Female	0.01	0.01 to 0.01	**<0.001**	0.00	0.00 to 0.00	**0.002**	‒0.00	‒0.00 to 0.00	0.806	‒0.01	‒0.01 to ‒0.01	**<0.001**
Neurological dysfunction	0.01	0.00 to 0.01	**<0.001**	0.01	0.01 to 0.02	**<0.001**	0.00	0.00 to 0.01	**0.019**	0.00	0.00 to 0.01	**0.016**
Creatinine >200 mmol/l	0.05	0.05 to 0.05	**<0.001**	0.02	0.01 to 0.02	**<0.001**	0.16	0.16 to 0.16	**<0.001**	0.02	0.02 to 0.03	**<0.001**
Recent MI	0.01	0.00 to 0.01	**<0.001**	0.01	0.00 to 0.01	**<0.001**	0.00	‒0.00 to 0.00	0.150	0.00	0.00 to 0.01	**<0.001**
Chronic pulmonary disease	0.01	0.01 to 0.01	**<0.001**	‒0.00	‒0.00 to 0.00	0.784	0.01	0.00 to 0.01	**<0.001**	‒0.00	‒0.00 to 0.00	0.381
Unstable angina	0.00	‒0.00 to 0.00	0.255	0.00	‒0.00 to 0.01	0.201	‒0.00	‒0.00 to 0.00	0.978	‒0.00	‒0.00 to 0.00	0.507
NYHA IV	0.01	0.01 to 0.01	**<0.001**	0.00	0.00 to 0.00	**<0.001**	0.01	0.01 to 0.01	**<0.001**	0.00	0.00 to 0.00	**<0.001**
Pulmonary HTN	0.03	0.02 to 0.03	**<0.001**	0.00	‒0.00 to 0.01	0.657	0.03	0.02 to 0.03	**<0.001**	0.01	0.00 to 0.01	**<0.001**
Diabetes on Insulin	0.01	0.01 to 0.01	**<0.001**	0.00	0.00 to 0.01	**0.006**	0.03	0.03 to 0.03	**<0.001**	‒0.00	‒0.01 to ‒0.00	**0.026**
Reference = good LV (EF > 50%)												
Very poor EF (EF < 20%)	0.03	0.02 to 0.03	**<0.001**	‒0.01	‒0.02 to 0.00	0.238	0.01	‒0.00 to 0.02	0.142	0.00	‒0.01 to 0.01	0.982
Poor EF (21–30%)	0.02	0.02 to 0.02	**<0.001**	‒0.00	‒0.01 to 0.01	0.916	0.02	0.01 to 0.02	**<0.001**	0.00	‒0.00 to 0.01	0.712
Moderate EF (31–50%)	0.00	0.00 to 0.01	**<0.001**	0.00	0.00 to 0.01	**<0.001**	0.00	0.00 to 0.00	**0.010**	0.00	‒0.00 to 0.00	0.083
Peripheral vascular disease	0.01	0.01 to 0.01	**<0.001**	0.02	0.02 to 0.02	**<0.001**	0.01	0.01 to 0.01	**<0.001**	0.00	‒0.00 to 0.00	0.093
Other than isolated CABG	0.00	‒0.00 to 0.00	0.088	0.01	0.00 to 0.01	**<0.001**	0.00	‒0.00 to 0.00	0.341	0.02	0.01 to 0.02	**<0.001**
Smooth function (day of the week) significance			*P* = 0.081			*P* = 0.137			*P* = 0.732			***P* = 0.039**
Observations			369 689			323 528			320 604			371 500
*R* ^2^			0.017			0.016			0.051			0.011

CABG: coronary artery bypass grafting; CI: confidence interval; NYHA: New York Heart Association. If *P*-value is significant (e.g. <0.05) it is bold.

**Figure 4:**
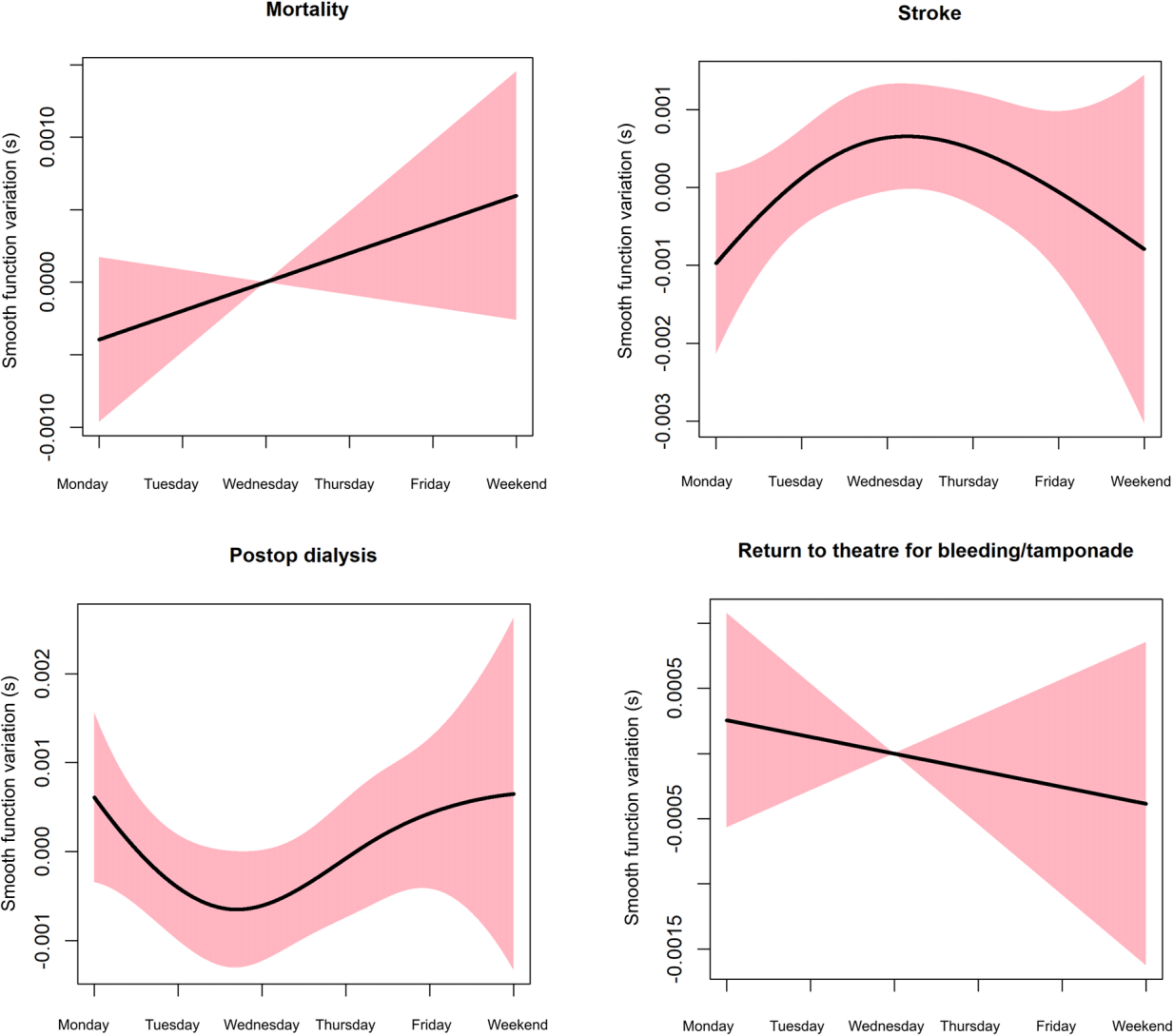
Generalized additive model plots to show the association between the morality, stroke need for pos-top dialysis and return to theatre for bleeding tamponade for the coronary artery bypass grafting subgroup.

Supplementary Material, Tables S3 and S4 summarize the baseline characteristics and crude outcomes of the isolated CABG group. After adjustment, there was no evidence that mortality (*P* = 0.164), postoperative CVA (*P* = 0.173), need for postoperative dialysis (*P* = 0.263) and return to theatre for bleeding (*P* = 0.534) differed across days of the week (Table 4). The smooth curves for these outcomes are summarized in Fig. 4.

**Table 4:** Effect estimates for the EuroSCORE linear predictor and the smooth function (day of the week) for the coronary artery bypass grafting subgroup

Predictors	Mortality			Postoperative CVA			Postoperative dialysis			Return to theatre for bleeding/tamponade		
	Estimates	CI	*P*-Value	Estimates	CI	*P*-Value	Estimates	CI	*P*-Value	Estimates	CI	*P*-Value
(Intercept)	−0.00	−0.05 to 0.04	0.911	−0.06	−0.14 to 0.02	0.164	0.00	−0.06 to 0.06	0.963	−0.01	−0.08 to 0.06	0.781
Age	0.00	0.00 to 0.00	**<0.001**	0.00	0.00 to 0.00	**<0.001**	0.00	0.00 to 0.00	**<0.001**	0.00	0.00 to 0.00	**<0.001**
Female	0.00	0.00 to 0.01	**<0.001**	0.00	0.00 to 0.00	**0.036**	−0.00	−0.00 to 0.00	0.658	−0.01	−0.01 to −0.00	**<0.001**
Neurological dysfunction	−0.01	−0.02 to 0.01	0.300	0.01	−0.02 to 0.04	0.592	−0.02	−0.04 to 0.01	0.180	−0.00	−0.03 to 0.02	0.692
Creatinine >200 mmol/l	0.01	0.00 to 0.01	**<0.001**	0.02	0.01 to 0.02	**<0.001**	0.00	0.00 to 0.01	**0.035**	0.00	−0.00 to 0.01	0.098
Recent MI	0.03	0.03 to 0.04	**<0.001**	0.02	0.01 to 0.02	**<0.001**	0.16	0.15 to 0.16	**<0.001**	0.01	0.00 to 0.02	**<0.001**
Chronic pulmonary disease	0.01	0.00 to 0.01	**<0.001**	0.01	0.00 to 0.01	**<0.001**	0.00	−0.00 to 0.00	0.052	0.01	0.00 to 0.01	**<0.001**
Unstable angina	0.01	0.01 to 0.01	**<0.001**	0.00	−0.00 to 0.00	0.922	0.00	0.00 to 0.01	**<0.001**	−0.00	−0.00 to 0.00	0.648
NYHA IV	0.00	0.00 to 0.01	**<0.001**	0.00	0.00 to 0.01	**0.003**	0.00	−0.00 to 0.00	0.050	0.00	−0.00 to 0.00	0.183
Pulmonary HTN	0.00	0.00 to 0.00	**<0.001**	0.00	0.00 to 0.00	**<0.001**	0.00	0.00 to 0.01	**<0.001**	−0.00	−0.00 to 0.00	0.209
Diabetes on Insulin	0.02	0.02 to 0.03	**<0.001**	−0.00	−0.02 to 0.01	0.454	0.00	−0.01 to 0.01	0.365	0.02	0.01 to 0.03	**0.001**
Reference = good LV (EF > 50%)												
Very poor EF (EF < 20%)	0.03	0.02 to 0.04	**<0.001**	−0.01	−0.02 to 0.00	0.080	0.01	−0.00 to 0.02	0.143	0.00	−0.01 to 0.01	0.760
Poor EF (21–30%)	0.02	0.01 to 0.02	**<0.001**	0.00	−0.01 to 0.01	0.664	0.01	0.01 to 0.02	**<0.001**	0.00	−0.00 to 0.01	0.262
Moderate EF (31–50%)	0.00	0.00 to 0.01	**<0.001**	0.00	0.00 to 0.01	**0.001**	0.00	−0.00 to 0.00	0.190	0.00	−0.00 to 0.00	0.081
Peripheral vascular disease	−0.03	−0.04 to −0.02	**<0.001**	0.01	−0.00 to 0.02	0.080	−0.01	−0.02 to 0.00	0.143	−0.00	−0.01 to 0.01	0.760
Other than isolated CABG	0.01	0.01 to 0.01	**<0.001**	0.02	0.01 to 0.02	**<0.001**	0.01	0.01 to 0.01	**<0.001**	0.00	−0.00 to 0.00	0.616
Smooth term (dayname)			0.164			0.173			0.263			0.534
Observations			237 716			208 060			205 379			238 812
*R* ^2^			0.013			0.018			0.060			0.008

CABG: coronary artery bypass grafting; CI: confidence interval; NYHA: New York Heart Association. If *P*-value is significant (e.g. <0.05) it is bold.

## DISCUSSION

In our analysis, we found no evidence that there was a variation in the incidence of postoperative mortality, stroke or need for dialysis after adult cardiac surgery. To our knowledge, this is the largest analysis examining the outcomes of cardiac surgery by day of the week and is reflective of real-world practice. Our results are in contrast to Aylin *et al.* [5] study that shows a higher risk of death in patients that have any type of elective surgical procedures later in the week or the weekend in the UK but similar to another study in Canada by Dubois *et al.* [2] showing no weekday effect on any type of elective surgery. Of note, unlike the current study, Aylin *et al.* included all types of elective surgery, including non-cardiac. Similar to our study, Dalén *et al.* found no weekend effect on mortality after cardiac surgery performed in Sweden in a study that included both elective and emergency procedures. However, the confounding effects between elective and emergency procedures were not analysed and have been further addressed in this study.

Two studies focused solely on weekdays and outcomes of non-elective CABG surgery in the USA. Beller *et al.* [8] found no independent effect of weekend surgery on mortality, but the patients requiring weekend surgery had a higher-risk profile. Similarly, Panhwar *et al.* [9] found no weekday effect on risk-adjusted mortality between weekend and weekday patients but an excess of crude mortality over the weekend due to a sick weekend cohort of patients. These results are consistent with the findings of the elective CABG subanalysis we have performed.

It has also been hypothesized that the poorer outcomes of surgery could be related to the lack of staff resources towards the weekend. However, Aldridge *et al.* [12] showed no correlation of mortality risk for emergency admissions and weekend staff levels. Also, surgeon fatigue towards the end of the week was hypothesized to play a role in worse surgery outcomes [13]. As shown in our analysis, these factors above are unlikely to impact outcomes after cardiac surgery. Potential explanations for these findings in the UK are that there is a separate dedicated team for weekend elective work, so the volume of weekend emergency work does not influence them.

Moreover, UK cardiac surgery consultant contracts have an average of 2 operating days a week. Also, contracts comply with European Working Time Directive. In theory, these factors could reduce fatigue amongst junior doctors and surgeon fatigue should be mitigated.

We have also shown that therates of return to theatre for bleeding are lower in the weekend, perhaps suggesting a heightened awareness of surgeons about this complication. As shown earlier, this could also be explained by the lower-risk profile of these patients (selection bias) or fewer complex, combined procedures performed over the weekend (Fig. 2). Of note, in the subgroup analysis of isolated CABG, this effect was not significant. This suggests that the higher volume of valve surgery may be implicated in the higher rates of bleeding during the week compared to the weekend. We have also noted that the first operator was more often a consultant than a non-consultant grade for all procedures over the weekend compared to the weekday. This could be another explanation for the lower return to theatre rates over the weekend. In the subgroup analysis for CABG, where no difference in return to theatre for bleeding was noted, we found almost the same percentage of consultants as the first operator in the weekday compared to the weekend.

The lower the risk profile of patients operated electively at the weekend is also reflected in the trends in fewer events (mortality, stroke and return to theatre for bleeding) at the weekend compared to the weekday. This finding is similar to the Aylin *et al.* [5] study. A natural explanation for this finding is likely selection bias—surgeons select more straightforward cases to be operated on the weekend initiative list.

### Limitations

Analysis of seasonality of outcomes implies handling complex non-linear interactions. Hence, we decided to use the GAM that could handle this type of interaction. Nevertheless, 1 disadvantage of GAMs is the high propensity to overfit. The analysis of the National Adult Cardiac Surgery Audit dataset is limited by the retrospective analysis of prospectively collected data regardless of the robust adjustment we implemented. The adjustment was made according to EuroSCORE 2 variables; however, residual confounding could still remain. Specific limitations to this analysis are errors in data entry that are inherent to administrative databases or missing data. We had no data on the longer-term outcomes of patients operated by day of the week in the UK. This was analysed by Dalén *et al.* [10], who found no weekday effect of cardiac surgery during 15 years of follow-up in a Swedish population.

## CONCLUSIONS

Our analysis found no evidence of a difference in risk-adjusted short-term mortality, stroke, and the requirement for postoperative dialysis across days of the week in patients undergoing any elective cardiac surgery. However, we found evidence of a reduced rate of return to theatre for bleeding for all procedures at the weekend. There was no day of the week variation in any outcome following elective isolated CABG. We conclude that it is safe to schedule/perform elective cardiac surgery on any day of the week.

## SUPPLEMENTARY MATERIAL

Supplementary material is available at *EJCTS* online.

## Supplementary Material

ezac038_supplementary_data

## Data Availability

The data underlying this article will be shared on reasonable request to the corresponding author.

## ABBREVIATIONS

CABG: Coronary artery bypass grafting
GAM: Generalized additive model
IQR: Interquartile range
MVR: Mitral valve repair/replacement
