# Supplementary material for: Weekday and outcomes of elective cardiac surgery in the UK: a large retrospective database analysis
Source: Eur J Cardiothorac Surg. 2022 Jan 29;61(6):1381–8. doi: 10.1093/ejcts/ezac038 (PMC9746893; doi:10.1093/ejcts/ezac038)
Supplement: ezac038_supplementary_data [file ezac038_supplementary_data.docx]

**Supplementary material**

**Weekday and Outcomes of Elective Cardiac Surgery in the UK: A Large Retrospective Database Analysis**

**Fudulu et al.**

Table 1 Diagnostics the fitted gam model for all procedures

| Outcome | edf | k-index | p-value |
| --- | --- | --- | --- |
| Mortality | 2.65 | 1 | 0.6 |
| Postoperative CVA | 4.2 | 1 | 0.48 |
| Need for postoperative dialysis | 1 | 1 | 0.50 |
| Return to theatre for bleeding | 2.75 | 1 | 0.3 |

Table 2 Diagnostics the fitted gam model for the isolated CABG group.

| Outcome | edf | k-index | p-value |
| --- | --- | --- | --- |
| Mortality | 2.65 | 1 | 0.6 |
| Postoperative CVA | 1.92 | 1.01 | 0.84 |
| Need for postoperative dialysis | 1 | 1 | 0.36 |
| Return to theatre for bleeding | 1 | 1 | 0.41 |

Table 3 Baseline characteristics of CABG patients operated by day of the week.

|  | **Monday (N= 50597)** | **Tuesday (N= 48106)** | **Wednesday (N= 48615)** | **Thursday (N= 45584)** | **Friday (N= 35545)** | **Weekend (N= 10365)** | **Overall (N= 238812)** |
| --- | --- | --- | --- | --- | --- | --- | --- |
| **Age (Mean, SD)** | 65.5 (9.30) | 65.6 (9.24) | 65.5 (9.31) | 65.5 (9.31) | 65.4 (9.35) | 65.6 (9.21) | 65.5 (9.30) |
| **Female** | 8919 (17.6%) | 8560 (17.8%) | 8551 (17.6%) | 7965 (17.5%) | 6279 (17.7%) | 1838 (17.7%) | 42112 (17.6%) |
| **Neurological Dysfunction** | 914 (1.8%) | 836 (1.7%) | 815 (1.7%) | 779 (1.7%) | 638 (1.8%) | 193 (1.9%) | 4175 (1.7%) |
| **Creatinine>200 µmol/L** | 684 (1.4%) | 607 (1.3%) | 650 (1.3%) | 618 (1.4%) | 485 (1.4%) | 141 (1.4%) | 3185 (1.3%) |
| **Recent MI** | 5007 (9.9%) | 4694 (9.8%) | 4728 (9.7%) | 4499 (9.9%) | 3414 (9.6%) | 952 (9.2%) | 23294 (9.8%) |
| **Pulmonary Disease** | 5401 (10.7%) | 5211 (10.8%) | 5188 (10.7%) | 4814 (10.6%) | 3902 (11.0%) | 1151 (11.1%) | 25667 (10.7%) |
| **CCS IV** | 3086 (6.1%) | 2846 (5.9%) | 2955 (6.1%) | 2663 (5.8%) | 2215 (6.2%) | 614 (5.9%) | 14379 (6.0%) |
| **NYHA IV** | 853 (1.7%) | 812 (1.7%) | 791 (1.6%) | 725 (1.6%) | 596 (1.7%) | 178 (1.7%) | 3955 (1.7%) |
| **Pulmonary HTN** | 275 (0.5%) | 196 (0.4%) | 265 (0.5%) | 178 (0.4%) | 193 (0.5%) | 47 (0.5%) | 1154 (0.5%) |
| **Diabetes on Insulin** | 3510 (6.9%) | 3363 (7.0%) | 3288 (6.8%) | 3129 (6.9%) | 2396 (6.7%) | 734 (7.1%) | 16420 (6.9%) |
| **LV function** |  |  |  |  |  |  |  |
| Very poor (EF<20%) | 132 (0.3%) | 150 (0.3%) | 133 (0.3%) | 107 (0.2%) | 101 (0.3%) | 25 (0.2%) | 648 (0.3%) |
| Poor (EF 21%-30%) | 515 (1.0%) | 528 (1.1%) | 501 (1.0%) | 517 (1.1%) | 385 (1.1%) | 103 (1.0%) | 2549 (1.1%) |
| Moderate (EF 31%-50%) | 4979 (9.8%) | 4898 (10.2%) | 4812 (9.9%) | 4518 (9.9%) | 3375 (9.5%) | 999 (9.6%) | 23581 (9.9%) |
| Good (EF>50%) | 44971 (88.9%) | 42530 (88.4%) | 43169 (88.8%) | 40442 (88.7%) | 31684 (89.1%) | 9238 (89.1%) | 212034 (88.8%) |
| **Peripheral Vascular Disease** | 6161 (12.2%) | 5862 (12.2%) | 5836 (12.0%) | 5376 (11.8%) | 4302 (12.1%) | 1273 (12.3%) | 28810 (12.1%) |
| **Consultant first operator** | 34507 (68.2%) | 32757 (68.1%) | 33207 (68.3%) | 31125 (68.3%) | 24252 (68.2%) | 7111 (68.6%) | 34507 (68.2%) |

Table 4 Crude outcomes of CABG patients operated by day of the week

|  | **Monday (N=50597)** | **Tuesday (N=48106)** | **Wednesday (N=48615)** | **Thursday**  **(N=45584)** | **Friday (N=35545)** | **Weekend (N=10365)** | **Overall (N=238812)** |
| --- | --- | --- | --- | --- | --- | --- | --- |
| **Mortality (30 day)** | 1330 (1.7%) | 1235 (1.6%) | 1225 (1.6%) | 1215 (1.7%) | 868 (1.6%) | 148 (1.1%) | 6021 (1.6%) |
| **Postop CVA** |  |  |  |  |  |  |  |
| TIA | 459 (0.6%) | 463 (0.6%) | 434 (0.6%) | 399 (0.6%) | 339 (0.6%) | 71 (0.5%) | 2165 (0.6%) |
| CVA with neurological recovery | 475 (0.6%) | 470 (0.6%) | 454 (0.6%) | 458 (0.6%) | 358 (0.7%) | 69 (0.5%) | 2284 (0.6%) |
| CVA with neurological deficit | 10158 (13.0%) | 10010 (13.0%) | 9564 (12.6%) | 9691 (13.5%) | 7348 (13.6%) | 1201 (8.6%) | 47972 (12.9%) |
| **Postoperative dialysis** | 1324 (1.7%) | 1386 (1.8%) | 1350 (1.8%) | 1292 (1.8%) | 928 (1.7%) | 204 (1.5%) | 6484 (1.7%) |
| **Return to theatre for bleeding/tamponade** | 2417 (3.1%) | 2451 (3.2%) | 2391 (3.1%) | 2267 (3.2%) | 1659 (3.1%) | 363 (2.6%) | 11548 (3.1%) |
